# Bibliographical

**Published:** 1858-12

**Authors:** 


### BIBLIOGRAPHICAL.

#### *Lallemand and Wilson on Spermatorrhea.*

We have received through the hands of Messrs. J. J. Richards & Co, Booksellers, of Atlanta, a work bearing the following title, "A Practical Treatise on the Causes, Symptoms and Treatment of Spermatorrhea, by M. Lallemand, Professor of Clinical Surgery, &c., &c." Translated by Henry J. McDougall, Third Am. Ed. To which is added "On Diseases of the Vesiculæ Seminales, and their Associated Organs, &c." By Morris Wilson, M. D. The work is published in the usual good taste and style of Messrs. Blanchard & Lea, Philadelphia.

"Of making many books there is no end." If this were true in the days of Solomon, it is eminently true in this age of ours, and of the many new books which demand the consideration of the Medical Profession, and justly so, we think this is one emphatically suited to the present time.

The subject of which it treats, is a most important one, and one that has been too long neglected, we may almost say, ignored by Medical writers and teachers. The mass of sufferers from Spematorrhea, in all its debilitating and destroying consequences to both mind and body, is, no doubt, astonishingly great, and hence, as true philanthropists, we gladly hail any light which may benefit the profession in alleviating the sufferings, and tend to free thousands, of *unfortunates* from the infamous thraldom of Charlatanism. Such we consider the work before us.

Whereas, we speak thus openly in commendation of this work, we do not mean to be understood as endorsing all it contains, or affirming that it embodies all that is beneficial or may be learned of the subject.

M. Lallemand has reasoned strictly on inductive principles in his investigations, giving a record of a great number of cases, and from the pathological phenomena afforded by these cases has drawn his conclusions and based his treatment.

He has treated largely of the *Causes* of this disease and has regularly classified them. He has given eight classes of causes, 1st Blennorrhagia; 2d, Cutaneous Affections; 3d, Irritation of the rectum; 4th, Acnae; 5th, Venereal Excesses; 6th, The Action of certain Remedies; 7th, The Action of the Cerebo-Spinal System, and 8th, Congenital Predisposition, and has adduced evidence from the cases he has given, to establish the justness of his conclusions.

Of his views of the symptoms of Spermatorrhea, we have only to say, they have at least the merit of symplicity, and are entitled to great weight from his extensive practice in this disease.

Of Dr. Wilson's Essay, we remark, that he has investigated and treated the subject as a Medical Philosopher should, and has stated his views concerning it fairly and plainly.

Of the comparative merit of the *Treatment*, as based upon the respective views, of M. M. Lallemand and Wilson, we have neither the time nor inclination to descant, nor is it expected that we should *review* a work in a Bibliographical notice. We say to all Physicians the book is published, buy it, study it—it will do you good.

We have also received from the same Publishers, Messrs. Blanchard & Lea, "Moreland on Urinary Organs." "Watson's Practice of Physic," Edited by Condie, and "Wilson's Human Anatomy, General and Special," Edited by Gobrecht, which will be noticed in the January number of the Journal.

